# Impact of Maternal Macronutrient Intake on Large for Gestational Age Neonates’ Risk Among Women with Gestational Diabetes Mellitus: Results from the Greek BORN2020 Cohort

**DOI:** 10.3390/nu17020269

**Published:** 2025-01-13

**Authors:** Antonios Siargkas, Antigoni Tranidou, Emmanuela Magriplis, Ioannis Tsakiridis, Aikaterini Apostolopoulou, Theodoros Xenidis, Nikolaos Pazaras, Michail Chourdakis, Themistoklis Dagklis

**Affiliations:** 1 3rd Department of Obstetrics and Gynecology, School of Medicine, Faculty of Health Sciences, Aristotle University of Thessaloniki, 541 24 Thessaloniki, Greece; antonis.siargkas@gmail.com (A.S.); antigoni.tranidou@gmail.com (A.T.); igtsakir@auth.gr (I.T.); xenidistheodoros@yahoo.com (T.X.); 2 Laboratory of Hygiene, Social & Preventive Medicine and Medical Statistics, School of Medicine, Faculty of Health Sciences, Aristotle University of Thessaloniki, 541 24 Thessaloniki, Greece; katapost@yahoo.gr (A.A.); nikospaz@hotmail.com (N.P.); mhourd@auth.gr (M.C.); 3 Department of Food Science and Human Nutrition, Agricultural University of Athens, Iera Odos 75, 118 55 Athens, Greece; emagriplis@eatsmart.gr

**Keywords:** maternal, diet, nutrition, pregnancy, macronutrients, carbohydrates, fat, protein, gestational diabetes mellitus, GDM, macrosomia, LGA

## Abstract

Background/Objectives: The effect of maternal macronutrient composition on the risk of large for gestational age (LGA) neonates among women with gestational diabetes mellitus (GDM) is not well understood. This study aimed to investigate these associations in a pregnant cohort in Northern Greece, considering both pre-pregnancy and early pregnancy dietary intake, and stratifying women by pre-pregnancy body mass index (BMI). Methods: From a total of 797 eligible pregnant women, the 117 (14.7%) who developed GDM (and thus were included in the study) completed the validated Food Frequency Questionnaires (FFQs). Macronutrient intake was assessed for the six months before pregnancy and until mid-gestation, prior to the oral glucose tolerance test. Data were compared with European Food Safety Authority (EFSA) guidelines, and participants were stratified by pre-pregnancy BMI (normal vs. overweight/obese). Multivariate logistic regression was used to estimate adjusted odds ratios (aORs) for LGA risk. Results: In normal-BMI women with GDM, higher dietary fiber (aOR = 1.39) and vegetable protein (aOR = 1.61) intake before pregnancy were both significantly associated with an increased risk of LGA. During early pregnancy, the elevated risk from vegetable protein persisted (aOR = 1.51). Among overweight/obese women, no significant pre-pregnancy associations were observed. However, during early pregnancy, a higher percentage of total carbohydrate intake was linked to increased LGA risk (aOR = 1.11), while maintaining saturated fatty acids “as low as possible” reduced the odds of LGA (aOR = 0.71). Elevated vegetable protein intake also increased LGA risk (aOR = 1.61). Conclusions: Maternal macronutrient intake prior to and during early pregnancy may influence LGA risk in GDM, with distinct patterns according to pre-pregnancy BMI. These findings underscore the importance of tailoring dietary recommendations—especially regarding fiber, vegetable protein, carbohydrates, and saturated fat—to mitigate the risk of LGA in women with GDM.

## 1. Introduction

Gestational diabetes mellitus (GDM) is a common pregnancy-related complication, associated with an increased risk of adverse perinatal outcomes, most notably the birth of large for gestational age (LGA) neonates [1]. Beyond the immediate risks to the infant, the presence of an LGA neonate in women with GDM may have long-term implications for the offspring’s metabolic health, potentially increasing their risk of developing obesity later in life [2]. However, the impact of GDM on LGA varies considerably across populations, with studies often yielding inconsistent results [3,4]. These variations may be influenced by population-specific factors, such as maternal dietary patterns, macronutrient composition, and lifestyle habits, which could play a significant role in shaping obstetric outcomes among women with GDM [5].

Expanding on these considerations, studies emphasize the importance of optimizing maternal health during the periconceptional period, a pivotal time for both fetal development and long-term health [6]. This period is regarded as a key window of opportunity, with maternal diet playing a central role in shaping pregnancy outcomes. Notably, the influence of nutrition appears to extend beyond the periconceptional period, impacting outcomes from preconception period and potentially throughout the early pregnancy period and even later [7]. While recent studies have increasingly focused on the role of maternal diet during pregnancy, the specific effects of macronutrient composition on perinatal outcomes, such as the risk of LGA in women with GDM, remain underexplored. Most research has centered on overall caloric intake and glycemic control, with relatively few studies investigating how the balance of macronutrients—carbohydrates, proteins, and fats—shapes outcomes like LGA risk [8].

In Greece, the traditional Mediterranean diet has been found to confer protective effects against GDM risk when adhered to for up to six months prior to pregnancy, while a high intake of fresh and processed meat has been associated with inverse results [9]. Despite the apparent benefits of the Mediterranean diet, there is a paucity of research focusing on the specific macronutrient composition that may be most advantageous for pregnant women with GDM. Furthermore, the Mediterranean diet itself can vary significantly in its composition, and its overall benefits may not adequately address the precise macronutrient balance required by this particular group [10]. This underscores the need for more targeted studies to identify optimal macronutrient ratios—carbohydrates, proteins, and fats—that could improve outcomes for women with GDM. Therefore, further research is needed to clarify these associations, particularly in the settings of a Greek pregnant cohort. Birth weight, a critical indicator of neonatal health and a predictor of future metabolic and developmental outcomes, serves as a key measure for exploring these relationships, as it is closely linked to maternal dietary intake, particularly in women with GDM [11].

This study aims to examine the relationship of maternal macronutrient intake before and during pregnancy with the incidence of LGA neonates among women with GDM. The analysis will account for various confounding factors and will stratify the data by body mass index (BMI) to provide more nuanced insights.

## 2. Materials and Methods

### 2.1. Study Design and Participants

This study is part of the ongoing BORN2020 epidemiological study in Northern Greece, which collects detailed information on dietary habits and physical characteristics of pregnant women. Participants were recruited from the 3rd Department of Obstetrics and Gynecology, School of Medicine, Faculty of Health Sciences Aristotle University of Thessaloniki, Greece. For this analysis, women with singleton pregnancies and a confirmed diagnosis of GDM were included. Eligibility criteria required participants to be over 18 years of age and to speak Greek fluently. Women with pre-existing diabetes or those following special diets due to health conditions (e.g., inflammatory bowel disease, celiac disease) were excluded.

### 2.2. Bioethics Committee

The study was approved by the Bioethics Committee at Aristotle University of Thessaloniki, Greece, prior to initiation (6.231/29 July 2020). All participants gave written informed consent to participate.

### 2.3. Dietary Assessment

Maternal dietary intake was evaluated using a Food Frequency Questionnaire (FFQ), validated for the Greek population, and administered via oral interviews conducted by trained personnel at two distinct time points, period A and period B [12]. Macronutrient data assessed pre-pregnancy intake for up to six months prior to pregnancy (Period A), and were collected during the first trimester at first perinatal visit, during 11 + 0 to 13 + 6 gestational weeks. Additionally, the macronutrient intake of early pregnancy up to 21–23 weeks and before the routine oral glucose tolerance test (OGTT) was assessed to evaluate pregnancy intake before GDM diagnosis.

Macronutrient intake (carbohydrates, proteins, fats) was calculated using a dietary analysis software for Windows (Nutrisurvey V. 2007). Nutrient intakes were averaged and adjusted for total energy intake. Intakes were compared against the recommended European Food Safety Authority (EFSA) guidelines. Macronutrient intake included total carbohydrates, fats, and proteins expressed as a percentage of total energy intake (E%) according to the EFSA dietary recommendation values. Energy intake from each macronutrient was calculated using standard values: 4 kcal per gram for carbohydrates and proteins and 9 kcal per gram for fats. Fat intake was further subdivided into monounsaturated fatty acids (MUFA) and polyunsaturated fatty acids (PUFA). Trans fatty acid (TFA) findings were not included in this in accordance with established dietary policies, which align with European guidelines aimed at minimizing TFA across the Greek population, including pregnant women [13]. The fat variables were further categorized into specific subgroups, including saturated fatty acids (SFA), MUFA, and PUFA. Additionally, eicosapentaenoic acid (DHA), while not classified as a macronutrient, was included in the analysis due to its relevance as part of the overall fatty acid intake, which may influence both maternal and fetal health.

### 2.4. Clinical and Anthropometric Data

Maternal clinical data, including pre-pregnancy BMI, maternal age, and GDM diagnosis, were collected through the interviews. GDM was diagnosed according to the criteria set by the Hellenic Society of Obstetricians and Gynecologists; following the results of the HAPO study, all pregnant women in Greece undergo screening for GDM at 24–28 weeks, using three measurements, fasting (<92 mg/dL) and at 60 min (<180 mg/dL) and 120 (<153 mg/dL) following the consumption of 75 g of glucose. If at least one of the measurements is equal to or above the corresponding cutoff, GDM is diagnosed [14].

### 2.5. Perinatal Outcome

The key perinatal outcome was LGA, defined as a birth weight above the 90th percentile, using standardized growth charts [15].

### 2.6. Subgroup Analysis

Based on their reported pre-pregnancy BMI, women were categorized into normal BMI (18.5–24.9 kg/m^2^) and high BMI, including overweight and obese women (≥25 kg/m^2^) according to standard classification criteria [16]. Only two individuals with GDM were borderline underweight and thus were included in the normal-weight category to ensure an adequate sample size for meaningful analysis. Pre-pregnancy weight was directly measured during the first interview conducted between 11 + 0 to 13 + 6 weeks of gestation. Given that this weight measurement was taken early in the pregnancy, we hypothesized that there had not been significant changes in weight between conception and the first trimester.

### 2.7. Statistical Analysis

Descriptive statistics were calculated to summarize demographic and clinical characteristics. Continuous variables with a normal distribution were expressed as means with standard deviations, while non-normally distributed variables were presented as medians with interquartile ranges (IQRs). Categorical variables were expressed as frequencies and percentages. Multivariable logistic regression models were employed to investigate the relationship between maternal macronutrient intake and perinatal outcomes, adjusting for confounding factors such as energy, maternal age, physical activity (times walking in a day), weight gain until 21–23 weeks of gestation as a continuous variable (determined by subtracting the pre-pregnancy baseline weight, as recorded in clinical records, from the weight measured at the 21–23 week visit), supplementation intake as a binary variable (including iron, calcium), smoking, parity, assisted reproductive technologies (ARTs) methods, and thyroid status. A *p*-value of <0.05 was considered statistically significant. All statistical analyses were conducted using the R programming language (v4.2.1).

Confounding factors were carefully selected based on their potential to influence both maternal dietary intake and the risk of LGA neonates, particularly among women with GDM. Energy intake was included to control for total caloric consumption, which can directly affect fetal growth and birth weight. Maternal age was considered due to its association with increased risk of GDM and its potential impact on pregnancy outcomes. Physical activity was included as it can influence gestational weight gain and overall health, with higher physical activity levels linked to a lower risk of adverse pregnancy outcomes. Gestational weight gain up to 21–23 weeks was included because it is a known predictor of birth weight and fetal growth. Supplementation intake was treated as a binary variable (yes/no), accounting for whether participants used any supplements during pregnancy, as these could affect maternal and fetal health and, in turn, birth weight outcomes. Smoking was considered as it is a well-established risk factor for poor pregnancy outcomes, including both growth restrictions and LGA. Parity was included because it has been shown to influence pregnancy outcomes, with the number of previous pregnancies affecting both fetal growth and the risk of complications like GDM. Additionally, ARTs were accounted for, as ART pregnancies may have distinct risk profiles for adverse outcomes compared to spontaneous pregnancies. Lastly, thyroid status was included due to the impact that thyroid disorders can have on maternal metabolism and fetal growth, which may affect birth weight.

## 3. Results

**Figure 1:**
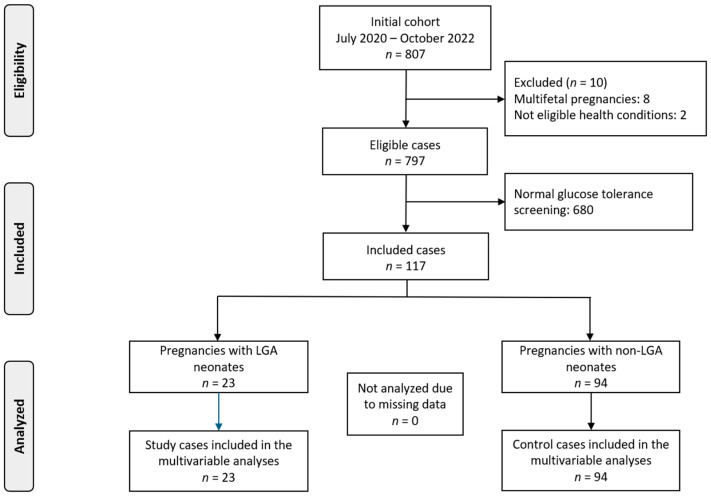
Flowchart.

The initial cohort included 797 pregnant women, among which 117 developed GDM and were included in the analysis. Those pregnancies that delivered LGA neonates served as our study group (*n* = 23) while the rest were our control group (*n* = 94), according to Figure 1. The analysis was stratified by BMI status, categorizing women as either normal BMI or high BMI. Table 1 shows the characteristics of the population under study.

### 3.1. LGA Risk in Normal-BMI Women

Table 2 presents the associations between macronutrient intake and LGA risk in normal-BMI women that developed GDM. Pre-pregnancy, a significant positive association was observed for dietary fiber intake, where women meeting or exceeding the adequate intake (AI) of 25 g/day had a 39% higher odds of having an LGA infant compared to those below the reference value (aOR = 1.39, 95% CI: 1.11–1.85, *p* = 0.008). Similarly, higher vegetable protein intake was significantly associated with increased LGA risk (aOR = 1.61, 95% CI: 1.20–2.44, *p* = 0.006).

During the first half of pregnancy, the association between dietary fiber intake remained increased but was not statistically significant (aOR = 1.27, 95% CI: 1.01–1.68, *p* = 0.057), suggesting a trend towards increased LGA risk with higher fiber consumption. Additionally, vegetable protein intake continued to show a significant positive association with LGA risk (aOR = 1.51, 95% CI: 1.14–2.17, *p* = 0.01). No other macronutrients, including total energy intake, were significantly associated with LGA risk in either period for normal-weight women with GDM.

### 3.2. LGA Risk in High-BMI Women

Table 3 outlines the associations between macronutrient intake and LGA risk in overweight and obese GDM women, for periods A and B. During the pre-pregnancy phase, none of the macronutrients examined were significantly associated with the risk of delivering an LGA infant. During period B, several significant associations emerged. A higher intake of total carbohydrates as a percentage of energy was associated with an 11% increase in the odds of having an LGA infant (aOR = 1.11, 95% CI: 1.01–1.26, *p* = 0.04). Maintaining SFA intake “as low as possible” during pregnancy was linked to a 29% reduction in LGA risk (aOR = 0.71, 95% CI: 0.52–0.90, *p* = 0.014). Furthermore, increased vegetable protein intake was significantly associated with 61% higher odds of delivering an LGA infant (aOR = 1.61, 95% CI: 1.15–2.68, *p* = 0.018). No other macronutrients demonstrated significant associations with LGA risk in the overweight and obese GDM cohort during pregnancy. Table S1 presents the post hoc power analysis for normal weight women, and Table S2 presents the post hoc power analysis for overweight and obese women.

## 4. Discussion

### 4.1. Main Findings

Our findings indicate that, in normal-BMI women, higher fiber and vegetable protein intake before and during early pregnancy correlates with an increased risk of delivering an LGA infant, whereas in overweight and obese women, a higher percentage of total carbohydrates and elevated vegetable protein intake during early pregnancy are associated with greater LGA risk, while a minimum intake of saturated fatty acids appears protective.

### 4.2. Interpretation of Our Findings

Although research on how pre-pregnancy dietary intake influences LGA risk is limited, a growing body of evidence underscores the role of maternal diet in shaping fetal growth and birth outcomes, with balanced nutrient intake shown to significantly affect birth size [17,18]. Our findings add to this understanding by highlighting how both the timing of dietary intake—pre-pregnancy versus early pregnancy—and maternal BMI can distinctly alter LGA risk among GDM women. Although many studies have focused on Western populations, cross-cultural evidence consistently links diets rich in nutrient-dense foods such as fruits, vegetables, and lean proteins to normal birth weights.

However, the influence of specific macronutrients, particularly different protein and fat sources, remains complex in women with GDM. Notably, our results challenge the assumption that higher fiber intake is invariably protective. In normal-BMI women, increased fiber and elevated vegetable protein intake were both associated with a greater likelihood of LGA. Similar patterns emerged for overweight and obese women during early pregnancy—though not before pregnancy—suggesting that the type and context of dietary choices may matter more than the simple categorization of foods as “high fiber” or “plant-based”. One explanation is that some high-fiber foods also contain substantial carbohydrates, leading to elevated glycemic loads if consumed in large quantities, while certain vegetable proteins (e.g., legumes accompanied by refined carbohydrates) may further raise postprandial glucose levels. Additionally, the general tendency of pregnant women to consume large amounts of fruits and vegetables could contribute to higher overall glycemic exposure in this population [19]. These findings align with the limited existing studies that have investigated the relationship between macronutrient intake before and during pregnancy and increased birth weight. A multiethnic Asian cohort study of 923 infants demonstrated that a pre-pregnancy diet rich in vegetables and fruits increased the risk of LGA neonates (RR: 1.31; 95% CI: 1.06, 1.62) [20]. Similarly, a study of 911 infants in China found that women with greater adherence to a “cereals–vegetables–fruits” diet before pregnancy had a higher risk of macrosomia (aOR: 2.22; 95% CI: 1.02, 4.84) [21]. On the other hand, a recent systematic review and meta-analysis reported no clear associations between maternal dietary patterns and LGA risk, potentially reflecting heterogeneity in study designs, dietary assessment methods, and cultural contexts [22]. Further research, ideally in more diverse populations and with detailed dietary assessments, is necessary to unravel these intricate relationships.

In overweight and obese women, no significant macronutrient associations emerged pre-pregnancy, but the early pregnancy period revealed several key relationships. In addition to the previously mentioned associations between increased fiber and vegetable protein intake and a higher incidence of LGA neonates, a greater proportion of carbohydrates contributing to total energy intake was also linked to an elevated risk of LGA. This finding suggests that excess carbohydrate consumption can exacerbate hyperglycemia in GDM, fueling increased fetal glucose exposure and growth. This association is known, and a meta-analysis of 1985 pregnancies revealed that a diet low on carbohydrates and thus a reduced glycemic index can cut the risk of LGA neonates in half (RR = 0.52, 95% CI: 0.31, 0.89) [23]. On the other hand, maintaining SFA intake “as low as possible” during pregnancy appeared protective for LGA among women with GDM. Our results agree with the observations of a small study 77 pregnant women which noted an increased saturated fatty acid concentration in the serum of the LGA neonates [24]. This finding seems to be explained by a study on rats that demonstrated that a high content of saturated fat activates placental mTOR and eIF2alpha signaling and increases fetal growth [25].

### 4.3. Strengths and Limitations

This study’s strengths include its focus on detailed macronutrient analysis across both pre-pregnancy and pregnancy periods, offering valuable insights into how dietary timing may affect perinatal outcomes in women with GDM, and to our knowledge it is the first study that addresses this in the Greek population among women with GDM. In addition, the stratified approach by maternal pre-pregnancy weight status allows for targeted findings that can guide dietary recommendations for normal-weight and overweight/obese women separately.

However, the study also has limitations, including its relatively small sample size, which could affect the generalizability of findings, and reliance on self-reported dietary data, introducing potential recall bias. While we tried to adjust for gestational weight gain based on the Institute of Medicine guidelines [26], the adjustment led to extreme incidence and adjusted odds ratio values, indicating insufficient data to support stable estimates in the analysis. In addition, we omitted detailed analysis due to Greece’s national policy, which enforces strict limitations on TFA levels in food. This policy aims to reduce TFA intake across the population, making TFA exposure less relevant in our study cohort. Another limitation is that a few important confounding factors associated with LGA were not available to include in our analysis. This included family history of diabetes, previous GDM, and previous macrosomia. Furthermore, a limitation of this study is that it only considers maternal dietary intake during the preconception period and early pregnancy. However, fetal growth is influenced by maternal nutrition throughout the entire pregnancy. Future studies that incorporate dietary data from the entire pregnancy period may provide a more comprehensive understanding of how maternal diet influences fetal growth and the risk of LGA. Moreover, due to the design of the study and data availability, we did not assess BMR or body composition directly, that could further refine the understanding of metabolic risk on GDM. Future studies with larger cohorts will be essential to validate these findings and achieve more reliable adjustments.

## 5. Conclusions

Overall, this study underscores the complexity of macronutrient influences on LGA risk in GDM, with important variations by BMI category and timing of intake. Among normal-BMI women, higher fiber and vegetable protein consumption before and during early pregnancy unexpectedly correlated with greater odds of LGA. In contrast, overweight and obese women showed no pre-pregnancy associations but experienced an increase in LGA risk with a higher carbohydrate percentage during early pregnancy and reduced risk with lower saturated fat intake. These findings highlight the multifaceted interplay between diet composition, metabolic status, and fetal growth in GDM. Future research should aim to clarify the role of specific plant-based foods, carbohydrate quality, and optimal fat subtypes to guide precision nutrition strategies for both normal-weight and high-BMI women at risk for GDM.

## Figures and Tables

**Table 1:** Maternal characteristics of the included population. Descriptive statistics: mean (SD), percentiles, or proportion.

Maternal Characteristics	LGA (*n* = 23)	Non-LGA (*n* = 94)	*p*-Value
Maternal age (years)	33.17 ± 3.2	34.39 ± 4.72	0.24
Maternal age > 35 years (%)	7 (30.43%)	44 (46.81%)	0.24
BMI pre-pregnancy overweight (%)	6 (26.09%)	15 (15.96%)	0.83
BMI pre-pregnancy obese (%)	4 (17.39%)	21 (22.34%)	0.81
Thyroid disease (%)	4 (17.39%)	9 (9.57%)	0.48
Parity			
-0 (%)	8 (34.78%)	52 (55.32%)	0.13
-1 (%)	12 (52.17%)	32 (34.04%)	0.17
-2 (%)	3 (13.04%)	9 (9.57%)	0.91
Smoking (%)	3 (13.04%)	18 (19.15%)	0.7
Assisted reproductive technology (ART) (%)	2 (8.7%)	9 (9.57%)	1
Treated with diet	18 (78.26%)	74 (78.72%)	1
Treated with insulin	5 (21.74%)	20 (21.28%)	1

Abbreviations: BMI: body mass index; thyroid disease: includes hypothyroidism, Hashimoto’s thyroiditis, hyperthyroidism; ART: assisted reproductive technology.

**Table 2:** Maternal macronutrient intake and LGA risk in normal-weight GDM women for period A and period B.

	Period A			Period B		
Macronutrients	Reference Values	*p*-Value	aOR (95% CI)	Reference Values	*p*-Value	aOR (95% CI)
Energy (E)	Average energy requirements *	0.21	0.99 (0.99, 1)	Average energy requirements *	0.059	0.99 (0.99, 0.99)
Carbohydrates (absolute value)	-	0.18	1.01 (0.99, 1.04)	-	0.66	0.99 (0.96, 1.02)
Dietary Fiber	AI 25 g/day	0.008 **	1.39 (1.11, 1.85)	AI 25 g/day	0.057	1.27 (1.01, 1.68)
Total Carbohydrates %	45–60 E%	0.21	1.06 (0.96, 1.17)	45–60 E%	0.89	1 (0.91, 1.11)
Fats	-	0.13	0.94 (0.88, 1)	-	0.71	1.01 (0.94, 1.09)
Eicosapentaenoic acid, DHA	250 mg/day DHA + EPA	0.32	105.16 (0, 1.13 × 10^6^)	250 (+) 100–200 mg/day DHA + EPA	0.12	586.73 (0.09, 2.47 × 10^6^)
Saturated Fatty Acids (SFA)	AI ALAP	0.23	0.89 (0.74, 1.06)	AI ALAP	0.28	0.9 (0.74, 1.07)
Total Fat %	RI 20–35%	0.13	0.91 (0.8, 1.01)	RI 20–35%	0.66	0.97 (0.88, 1.07)
Protein	AR 0.66 g/kg bw per day	0.45	1.02 (0.95, 1.11)	AR (+) 0.52 + 7.2 g/kg bw per day (1st and 2nd trimester)	0.99	0.99 (0.92, 1.08)
Protein %	-	0.37	1.12 (0.86, 1.48)	-	0.73	1.04 (0.8, 1.33)
Vegetable Protein	-	0.006 **	1.61 (1.2, 2.44)	-	0.01 **	1.51 (1.14, 2.17)
Animal Protein	-	0.63	0.98 (0.9, 1.06)	-	0.3	0.95 (0.86, 1.03)
Mono-unsaturated Fatty Acids (MUFA)	-	0.23	0.95 (0.86, 1.02)	-	0.38	1.04 (0.94, 1.16)
Poly-unsaturated Fatty Acids (PUFA)	-	0.75	0.96 (0.71, 1.09)	-	0.24	1.2 (0.87, 1.66)

Abbreviations: AI, adequate intake; ALAP, as low as possible; AR, average requirement; E%, percentage of energy intake; LGA, large for gestational age; * Before pregnancy, the average energy requirements are 2147 kcal/day for women aged 18–29 and 2072 kcal/day for those aged 30–39. During pregnancy, these requirements increase to 2477 kcal/day for women aged 18–29 and 2402 kcal/day for women aged 30–39. ** denotes statistical significance

**Table 3:** Maternal macronutrient intake and LGA risk in overweight and obese GDM women for period A and period B.

	Period A			Period B		
Macronutrients	Reference Values	*p*-Value	aOR (95% CI)	Reference Values	*p*-Value	aOR (95% CI)
Energy (E)	Average energy requirements *	0.16	1 (0.99, 1)	Average energy requirements *	0.055	1 (1, 1)
Carbohydrates (absolute value in gr)	-	0.14	0.97 (0.93, 1)	-	0.17	1.01 (0.99, 1.04)
Dietary Fiber	AI 25 g/day	0.9	0.98 (0.79, 1.21)	AI 25 g/day	0.28	1.12 (0.91, 1.42)
Total Carbohydrates %	45–60 E%	0.11	0.91 (0.8, 1.01)	45–60 E%	0.04 **	1.11 (1.01, 1.26)
Fats	-	0.25	1.03 (0.97, 1.1)	-	0.15	0.96 (0.9, 1.01)
Eicosapentaenoic acid, DHA	250 mg/day DHA + EPA	0.22	111.31 (0.07, 4.89 × 10^5^)	250 (+) 100–200 mg/day DHA + EPA	0.65	0.07 (6.08 × 10^−7^, 3809.12)
Saturated Fatty Acids (SFA)	AI ALAP	0.091	1.12 (0.98, 1.3)	AI ALAP	0.014 **	0.71 (0.52, 0.9)
Total Fat %	RI 20–35%	0.22	1.05 (0.97, 1.16)	RI 20–35%	0.076	0.91 (0.8, 0.99)
Protein	AR 0.66 g/kg bw per day	0.58	1.02 (0.95, 1.1)	AR (+) 0.52 + 7.2 g/kg bw per day (1st and 2nd trimester)	0.99	1 (0.88, 1.13)
Protein %	-	0.42	1.12 (0.85, 1.51)	-	0.37	0.88 (0.65, 1.13)
Vegetable Protein	-	0.15	1.21 (0.94, 1.65)	-	0.018 **	1.61 (1.15, 2.68)
Animal Protein	-	0.89	1 (0.93, 1.08)	-	0.22	0.93 (0.82, 1.03)
Mono-unsaturated Fatty Acids (MUFA)	-	0.54	1.02 (0.93, 1.11)	-	0.33	0.96 (0.89, 1.03)
Poly-unsaturated Fatty Acids (PUFA)	-	0.81	0.97 (0.79, 1.15)	-	0.89	1.02 (0.75, 1.36)

Abbreviations: AI, adequate intake; ALAP, as low as possible; AR, average requirement; E%, percentage of energy intake; LGA, large for gestational age; * Before pregnancy, the average energy requirements are 2147 kcal/day for women aged 18–29 and 2072 kcal/day for those aged 30–39. During pregnancy, these requirements increase to 2477 kcal/day for women aged 18–29 and 2402 kcal/day for women aged 30–39. ** denotes statistical significance

## Data Availability

Data are unavailable due to privacy restrictions.

## Supplementary Materials

The following supporting information can be downloaded at: https://www.mdpi.com/article/10.3390/nu17020269/s1, Table S1: Post hoc power analysis for normal weight women; Table S2: Post hoc power analysis for overweight and obese women.
