# Supplementary material for: Impact of Maternal Macronutrient Intake on Large for Gestational Age Neonates’ Risk Among Women with Gestational Diabetes Mellitus: Results from the Greek BORN2020 Cohort
Source: Nutrients. 2025 Jan 13;17(2):269. doi: 10.3390/nu17020269 (PMC11767984; doi:10.3390/nu17020269)
Supplement: Supplementary file 1 [file nutrients-17-00269-s001.zip › nutrients-3382024-supplementary.pdf]

Table S1. Post hoc power analysis for normal weight women

Macronutrients	Power (aoR) Period A	Power (aoR) Period B
Energy (E)	0.05	0.05
Carbohydrates (absolute value)	0.051	0.05
Dietary Fiber	0.512	0.3
Total Carbohydrates %	0.066	0.05
Fats	0.061	0.051
Eicosapentaenoic acid, DHA	1	1
Saturated Fatty Acids (SFA)	0.098	0.091
Total Fat %	0.083	0.051
Protein	0.053	0.05
Protein %	0.11	0.057
Vegetable Protein	0.806	0.688
Animal Protein	0.052	0.059
Mono-unsaturated Fatty Acids (MUFA)	0.06	0.059
Poly-unsaturated Fatty Acids (PUFA)	0.056	0.198

Table S2. Post hoc power analysis for overweight and obese women

Macronutrients	Power (aoR) Period A	Power (aoR) Period B
Energy (E)	0.05	0.05
Carbohydrates (absolute value)	0.052	0.051
Dietary Fiber	0.05	0.087
Total Carbohydrates %	0.07	0.083

Fats	0.053	0.054
Eicosapentaenoic acid, DHA	1	1
Saturated Fatty Acids (SFA)	0.087	0.362
Total Fat %	0.058	0.073
Protein	0.051	0.05
Protein %	0.085	0.09
Vegetable Protein	0.157	0.622
Animal Protein	0.05	0.062
Mono-unsaturated Fatty Acids (MUFA)	0.052	0.053
Poly-unsaturated Fatty Acids (PUFA)	0.051	0.051
